# Increased infectivity in human cells and resistance to antibody-mediated neutralization by truncation of the SIV gp41 cytoplasmic tail

**DOI:** 10.3389/fmicb.2013.00117

**Published:** 2013-05-14

**Authors:** Takeo Kuwata, Takaki Kaori, Ikumi Enomoto, Kazuhisa Yoshimura, Shuzo Matsushita

**Affiliations:** ^1^Center for AIDS Research, Kumamoto UniversityKumamoto, Japan; ^2^AIDS Research Center, National Institute of Infectious DiseasesTokyo, Japan

**Keywords:** SIV, gp41, truncation, infectivity, resistance, neutralization, antibody

## Abstract

The role of antibodies in protecting the host from human immunodeficiency virus type 1 (HIV-1) infection is of considerable interest, particularly because the RV144 trial results suggest that antibodies contribute to protection. Although infection of non-human primates with simian immunodeficiency virus (SIV) is commonly used as an animal model of HIV-1 infection, the viral epitopes that elicit potent and broad neutralizing antibodies to SIV have not been identified. We isolated a monoclonal antibody (MAb) B404 that potently and broadly neutralizes various SIV strains. B404 targets a conformational epitope comprising the V3 and V4 loops of Env that intensely exposed when Env binds CD4. B404-resistant variants were obtained by passaging viruses in the presence of increasing concentration of B404 in PM1/CCR5 cells. Genetic analysis revealed that the Q733stop mutation, which truncates the cytoplasmic tail of gp41, was the first major substitution in Env during passage. The maximal inhibition by B404 and other MAbs were significantly decreased against a recombinant virus with a gp41 truncation compared with the parental SIVmac316. This indicates that the gp41 truncation was associated with resistance to antibody-mediated neutralization. The infectivities of the recombinant virus with the gp41 truncation were 7,900-, 1,000-, and 140-fold higher than those of SIVmac316 in PM1, PM1/CCR5, and TZM-bl cells, respectively. Immunoblotting analysis revealed that the gp41 truncation enhanced the incorporation of Env into virions. The effect of the gp41 truncation on infectivity was not obvious in the HSC-F macaque cell line, although the resistance of viruses harboring the gp41 truncation to neutralization was maintained. These results suggest that viruses with a truncated gp41 cytoplasmic tail were selected by increased infectivity in human cells and by acquiring resistance to neutralizing antibody.

## INTRODUCTION

The RV144 trial demonstrated 31% vaccine efficacy for preventing human immunodeficiency virus type 1 (HIV-1) infection (Rerks-Ngarm et al., 2009). Antibodies against the HIV-1, particularly against the V1/V2 loops, correlate inversely with infection risk (Haynes et al., 2012). Further recent isolation of monoclonal antibodies (MAbs) that neutralize a broad range of HIV-1 strains suggest the possibility for developing a vaccine that can induce cross-neutralizing antibodies effective for various HIV-1 strains (Kwong and Mascola, 2012). Although non-human primate models of simian immunodeficiency virus (SIV) infection can facilitate the evaluation of immunogens, epitopes and immune correlates, no potent and broad neutralizing MAb against SIV had been available.

To understand the mechanisms involved in neutralization of infectivity by antibodies in an SIV model, we recently isolated MAb B404 from a SIVsmH635FC-infected rhesus macaque, which potently and broadly neutralizes various SIV strains, such as SIVsmE543-3, SIVsmE660 and the neutralization-resistant variants, genetically diverse SIVmac316, and highly neutralization-resistant SIVmac239 (Kuwata et al., 2011). The B404 epitope, which comprises the V3 and V4 loops of Env and is intensely exposed by ligation of Env to CD4, is the target for potent and broad neutralization of SIV (Kuwata et al., 2013). Vigorous induction of B404-like neutralizing antibodies using the specific VH3 gene with a long complementarity-determining region 3 loop and λ light chain was observed in four SIVsmH635FC-infected macaques. The B404-resistant variants were induced by passaging viruses in the presence of increasing concentrations of B404. Genetical analysis of the gp120 region of B404-resistant variants revealed that the mutations in the C2 region of Env were important for the resistance to antibody-mediated neutralization (Kuwata et al., 2013).

In the present study, we further analyzed B404-resistant variants and determined the precise region responsible for the resistance to antibody-mediated neutralization. Genetic analysis of viruses during passage in the presence of B404 as well as phenotypic analysis using recombinant viruses revealed that a truncation of the gp41 cytoplasmic tail was the primary step leading to escape from neutralization.

## MATERIALS AND METHODS

### CELLS

PM1 (Lusso et al., 1995), PM1/CCR5 (Yusa et al., 2005), and HSC-F (Akari et al., 1999) cells were maintained in Roswell Park Memorial Institute (RPMI) 1640 medium containing 10% fetal bovine serum (FBS). TZM-bl (Platt et al., 1998; Derdeyn et al., 2000; Wei et al., 2002; Takeuchi et al., 2008) and 293T (DuBridge et al., 1987) cells were maintained in Dulbecco’s modified Eagle’s medium containing 10% FBS.

### GENETIC ANALYSIS OF B404-RESISTANT VARIANTS

The induction of variants resistant to Fab-B404 (Kuwata et al., 2011) from SIVmac316 (Mori et al., 1992) harboring full-length gp41 was performed as described previously (Yoshimura et al., 2006; Hatada et al., 2010; Kuwata et al., 2013). Briefly, 5,000 TCID_50_ (50% tissue culture infectious dose) SIVmac316 was incubated with 5 ng/ml Fab-B404 for 30 min at 37°C. Then, 5 × 10^4^ PM1/CCR5 cells were added to the virus–Fab mixture. After incubation for 5 h, cells were washed with phosphate-buffered saline (PBS) and resuspended in RPMI 1640 supplemented with 10% FBS without Fab-B404. The culture supernatant was harvested 7 days later and used to infect fresh PM1/CCR5 cells for the next round of culture in the presence of increasing concentrations of Fab-B404. Proviral DNA samples were extracted from cells using a QIAamp DNA Blood Mini Kit (QIAGEN, Hilden, Germany) after 8, 17, 20, 23, and 26 passages as well as from P26C cells obtained after 26 passages in the absence of Fab-B404. The gp120 region was amplified using Platinum Taq DNA Polymerase High Fidelity (Invitrogen, Carlsbad, CA, USA) with primers SEnv-F (5^′^-ATG GGA TGT CTT GGG AAT CAG C-3^′^) and SER1 (5^′^-CCA AGA ACC CTA GCA CAA AGA CCC-3^′^). The whole *env* gene was amplified with primers SRev-F (5^′^-GGT TTG GGA ATA TGC TAT GAG-3^′^) and SEnv-R (5^′^-CCT ACT AAG TCA TCA TCT T-3^′^). The polymerase chain reaction (PCR) products were cloned using a TA cloning kit (Invitrogen), and subjected to sequencing. Nucleotide sequences were aligned and analyzed phylogenetically using Molecular Evolutionary Genetics Analysis version 5 (MEGA5) (Tamura et al., 2011).

### CONSTRUCTION OF INFECTIOUS MOLECULAR CLONES WITH THE Env REGION FROM B404-RESISTANT VARIANTS

One of the clones from passage 26, P26B404 clone 26, was selected for construction of recombinant viruses, because this clone had mutations typical of the major population of P26B404 variants. Infectious molecular clones SS, SN, and NS were generated by replacing fragments *Sph*I–*Sac*I [nucleotides (nt) 6,446–9,226], *Sph*I–*Nhe*I (nt 6,446–8,742), and *Nhe*I–*Sac*I (nt 8,742–9,226) with the corresponding regions of SIVmac316, respectively. Mutants F277V and N295S, which have point mutations at amino acid residues 277 and 295 of Env, respectively, were constructed by PCR mutagenesis using the SIVmac316 plasmid as template. The changes from phenylalanine (TTC) to valine (GTC) in F277V and asparagine (AAT) to serine (AGT) in N295S were introduced using primers F277VFw (5^′^-TTG GTT TGG CGT CAA TGG TAC TAG GGC-3^′^), F277VRv (5^′^-GTA CCA TTG ACG CCA AAC CAA G-3^′^), N295SFw (5^′^-GGC AAT AGT AGT AGA ACC ATA ATT AG-3^′^), and N295SRv (5^′^-AAT TAT GGT TCT ACT ACT ATT GCC-3^′^). Mutant and parental SIVmac316 plasmids were transfected into 293T cells using X-tremeGENE 9 DNA Transfection Reagent (Roche Molecular Biochemicals, Mannheim, Germany). After 2 days, the supernatants containing viruses were filtered (0.45 μm) and stored at -80°C.

### ANALYSIS OF VIRAL INFECTIVITY

For determination of TCID_50_ in PM1 and PM1/CCR5 cells, 5 × 10^4^ cells in 50 μl were inoculated with 50 μl serially diluted virus stocks in a 96-well plate and cultured for 2 weeks. Virus replication was judged by observation of cytopathic effects (CPE) by light microscopy. The TCID_50_ in TZM-bl cells was determined by measuring luciferase activities. Briefly, 100 μl medium, 50 μl serially diluted virus stock, and 50 μl 1 × 10^4^ cells containing 37.5 μg/ml diethylaminoethyl (DEAE) dextran were added to the wells of a 96-well plate. The plate was then incubated at 37°C for 2 days. After washing with PBS, cells were lysed with 30 μl cell lysing buffer (Promega, Madison, WI, USA) for 15 min at room temperature (RT) and then 10 μl of cell lysate was transferred to a 96-well white solid plate (Coster, Cambridge, MA, USA). Luciferase activity was measured using a Centro XS3 LB960 microplate luminometer (Berthold Technologies, Bad Wildbad, Germany) and a luciferase assay system (Promega). The TCID_50_ was calculated according to the formula of Reed and Muench (1938).

Infectivity of viruses in PM1, PM1/CCR5, and HSC-F cells was evaluated by detecting infected cells using flow cytometry as described previously (Kuwata et al., 2011). Briefly, PM1 and PM1/CCR5 cells were adjusted to 1 × 10^6^ cells/ml and HSC-F cells were adjusted to 5 × 10^6^ cells/ml. Aliquots of 100 μl cells per well in a 24-well plate were inoculated with 100 μl of diluted virus stocks. After incubation for 6 h, 800 μl fresh medium was added to wells. One-half of the cells in each well were collected at 4, 7, and 10 days post-inoculation. Cells were washed with PBS and fixed with IC Fixation Buffer (eBioscience, San Diego, CA, USA). After washing with Permeabilization Buffer (eBioscience) twice, the cells were intracellularly stained with 4 μg/ml (50 μl) anti-p27 Fab, B450 (Kuwata et al., 2011) by incubation for 20 min at RT. The cells were then incubated with 50 μl anti-HA antibody (1:200; 3F10, Roche Molecular Biochemicals) for 20 min at RT followed by incubation with 50 μl of anti-rat-FITC (1:500; Santa Cruz Biotechnology, Santa Cruz, CA, USA) for 20 min at RT. The stained cells were analyzed using a FACSCalibur (BD Biosciences, Franklin Lakes, NJ, USA). Frequencies of infected cells were determined by comparison with an uninfected control. Data analysis was performed using FlowJo (TreeStar, San Carlos, CA, USA).

All infectivity experiments were performed at least twice and the representative results are shown.

### ANALYSIS OF NEUTRALIZING ACTIVITIES

The Fab clones B404 and K8, isolated from an SIV-infected macaque (Kuwata et al., 2011), and murine MAb M318T (Matsumi et al., 1995) were used to examine the sensitivity of viruses to antibody-mediated neutralization in TZM-bl cells as described previously (Kuwata et al., 2011). Briefly, 100 μl serially diluted antibodies in duplicate were incubated with 200 TCID_50_ (50 μl) of virus in a 96-well plate. After incubation for 1 h at 37°C, 100 μl of 1 × 10^5^ TZM-bl cells/ml containing 37.5 μg/ml DEAE dextran were added. After incubation for 2 days, luciferase activities were measured as described above for the analysis of viral infectivity. The 50% inhibitory concentrations (IC_50_) and maximal percent of inhibition (MPI) were calculated from the average values by non-linear regression using Prism5 (GraphPad Software, San Diego, CA, USA).

Sensitivity to neutralization by B404 in macaque cells was analyzed using HSC-F cells, a cynomolgus macaque cell line immortalized by infection with *Herpesvirus saimiri* (Akari et al., 1999). Fab-B404 was serially diluted and 50 μl aliquots were mixed with 50 μl undiluted or 10-fold diluted virus in a 96-well plate. After 1 h incubation at 37°C, 2 × 10^5^ cells in 100 μl were added to each well and cultured for 1 day. The infected cells were washed twice with PBS, resuspended in 200 μl fresh medium, and cultured in a new 96-well plate. Viral infection was examined 4 days post-inoculation by intracellular staining of p27, as described above for the analysis of viral infectivity. Infectivity was determined in duplicate and the average value was used for the analysis of neutralization.

All neutralizing assays were performed at least twice and the representative results are shown.

### WESTERN BLOTTING ANALYSIS OF VIRAL PROTEINS

Cells and supernatants were collected from six-well plate 2 days after transfection of 293T cells with infectious molecular clones, as previously described (Yuste et al., 2005). Supernatants were filtered (0.45 μm) and clarified by centrifugation for 10 min at 3,000 rpm. The clarified supernatants were centrifuged at 13,200 rpm for 90 min at 4°C, and the viral pellets were resuspended in 1 ml PBS and centrifuged again. Pellets were then dissolved in 80 μl sample buffer [62.5 mM Tris–HCl, pH 6.8, 2% sodium dodecyl sulfate (SDS), 25% glycerol, 5% 2-mercaptoethanol, 0.01% bromophenol blue]. Cells were washed with PBS and lysed in 300 μl sample buffer. Samples of virions and cell lysates were boiled for 5 min, and the proteins were separated by SDS-polyacrylamide gel electrophoresis using SuperSep Ace 5–20% (Wako Pure Chemical Industries, Osaka, Japan). Proteins were transferred to an Immun-Blot PVDF Membrane (Bio-Rad Laboratories, Hercules, CA, USA). The membrane was blocked with 5% skim milk TBS-T (Tris-buffered saline containing 0.1% Tween 20) for 1 h at RT, and then washed three times with TBS-T. For the detection of gp120, the membrane was incubated overnight at 4°C with 1 μg/ml M318T (Matsumi et al., 1995) in 5% skim milk TBS-T. After washing three times with TBS-T, the membrane was incubated with anti-mouse immunoglobulin G (IgG) peroxidase (1:4,000, Santa Cruz Biotechnology) for 1 h at RT. The membrane was washed three times with TBS-T and once with TBS, and then TMB solution (KPL, Gaithersburg, MD, USA) was added to develop color. Viral proteins gp41 and p26 were similarly examined using crude supernatants from bacterial culture producing B408 and B450 (Kuwata et al., 2011), which were mixed with the same amount of 5% skim milk TBS-T. The membrane was incubated with anti-HA-HRP antibody (1:1,000; Roche Molecular Biochemicals) and Chemi-Lumi One L (Nacalai Tesque, Kyoto, Japan), and viral proteins were visualized using ImageQuant LAS 4000 (GE Healthcare, Piscataway, NJ, USA)

## RESULTS

### EVOLUTION OF VIRUSES DURING PASSAGE UNDER THE PRESSURE OF Fab-B404

To select for variants resistant to MAb B404, an antibody that targets a conformational epitope comprising the gp120 V3 and V4 loops, we passaged SIVmac316 that possesses a full-length gp41 in PM1/CCR5 cells in the presence of increasing concentrations of Fab-B404. The virus recovered at passage 26 (P26B404) was resistant to neutralization by B404 (V3/V4) and other antibodies, MAbs K8 (CD4i) and M318T (V2), that target epitopes other than that recognized by B404 (Kuwata et al., 2013). The region covering the whole *env* gene were amplified by PCR and cloned from viruses at passage 8, 17, 20, 23, and 26. The nucleotide sequences were phylogenetically analyzed to show the evolution of B404-resistant variants (**Figure 1**). The first major mutation was a change from glutamine (CAG) to a stop codon (TAG) at 733rd amino acid residue of Env. The Q733stop substitution in the gp41 cytoplasmic domain was observed in 12 of 14 clones at passage 8 and in all clones thereafter. Another stop codon (W782stop) was the second major mutation, which was detected after 17 passages. Substitutions V17L in the signal peptide and E176K in the V2 loop emerged after 20 and 23 passages, respectively, although the E176K substitution was also observed in P26C, control viruses after 26 passages in the absence of B404 (**Table 1**). In addition to these substitutions, most of clones acquired the F277V substitution in the late stage of evolution, except for one group at passage 26 which has the N295S substitution (see **Figure 1**, group 26II). Group 26II was clearly distinguished from group 26I by amino acid substitutions, such as T136M, N295S, and D571M/E (**Table 1**), suggesting two lineages of variants in P26B404.

**FIGURE 2:**
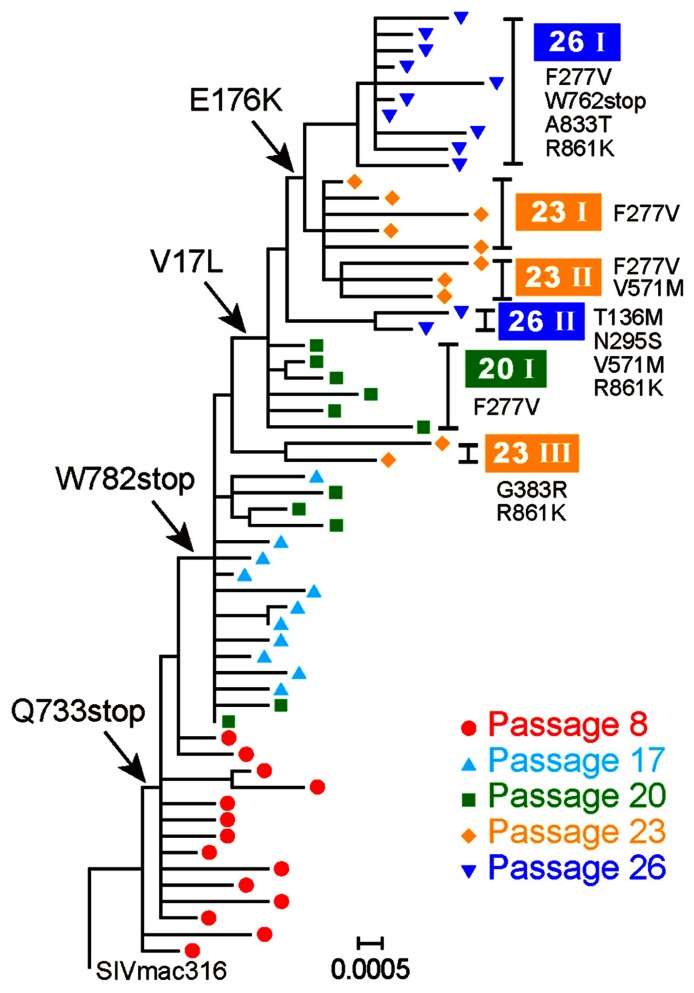
**Evolution of B404-resistant variants**. Nucleotide sequences of the *env* gene, which were derived from proviruses at passages 8, 17, 20, 23, and 26, were phylogenetically analyzed using MEGA5. Arrows indicate major substitutions as follows: Q733stop, W782stop, V17L, and E176K. Clones with representative amino acid substitutions were separated into groups, which are shown on the right side along with the corresponding substitutions.

**Table 1:** Frequency* of amino acid substitutions in Env clones from B404-resistant variants after 26 passages.

Substitution	Region	P26B404		P26C
		I	II
	gp120	(*n* = 22)	(*n* = 8)	(*n* = 14)
V17L	Signal peptide	**100%**	**100%**	0.0%
G62S	C1	0.0%	0.0%	21.4%
M67V/L/T	C1	4.5%	0.0%	21.4%
A68T	C1	0.0%	0.0%	**92.9%**
T136M	V1	4.5%	**87.5%**	0.0%
T137I	V1	0.0%	0.0%	14.3%
K141E/R	V1	0.0%	12.5%	7.1%
E176K	V2	**90.9%**	12.5%	35.7%
F277V	C2	**100%**	0.0%	0.0%
N295S	C2	0.0%	**100%**	0.0%
Q341H	V3	13.6%	12.5%	14.3%
D374N	C3	0.0%	0.0%	28.6%
K403R	V4	0.0%	12.5%	7.1%
W441R	C4	4.5%	0.0%	7.1%
**gp41**	**(*n* = 10)**	**(*n* = 2)**	**(*n* = 7)**
F528S/L	Extracellular	20.0%	0.0%	0.0%
D571M/E	Extracellular	10.0%	**100%**	0.0%
Q733stop	Cytoplasmic	**100%**	**100%**	0.0%
W762stop	Cytoplasmic	**100%**	0.0%	0.0%
W782stop	Cytoplasmic	**100%**	0.0%	0.0%
A833T	Cytoplasmic	**90.0%**	0.0%	0.0%
R839K	Cytoplasmic	0.0%	0.0%	**57.1%**
R861K	Cytoplasmic	**100%**	**100%**	0.0%

*Percentages of substitutions in populations P26B404 and P26C, which were obtained after 26 passages in the presence and absence of B404, respectively, are shown. The P26B404 population is separated into two subpopulations according to the phylogenetic analysis in *Figure 1*. All the substitutions that are observed in more than one clone are shown here. Boldface indicates substitutions dominant (>50%) in each population. The numbers of clones analyzed for the gp120 and gp41 regions are shown in parentheses.

These results demonstrated that the first step in acquiring resistance to B404 was the truncation of gp41. Although substitutions in gp120, represented by F277V, might contribute to the resistance to a high concentration of B404, 20 passages were required for the emergence of these substitutions.

### TRUNCATION OF gp41 CONFERRED RESISTANCE TO ANTIBODY-MEDIATED NEUTRALIZATION

To analyze effect of substitutions in B404-resistant variants on resistance to neutralization, recombinant viruses were constructed (**Figure 2**). The *env* region of SIVmac316 was replaced by that of P26B404 clone 26, which had substitutions typical to the P26I group. The resultant molecular clones SS, SN, and NS had substitutions in the entire *env* region, gp120 and gp41 from P26B404I, respectively. SS and NS were predicted to have a truncated gp41 with no other mutation in gp41, because the Q733stop substitution was the first substitution in gp41. Point mutants with substitutions F277V and N295S, which were representative mutations at late passages, were also constructed by PCR mutagenesis.

**FIGURE 2:**
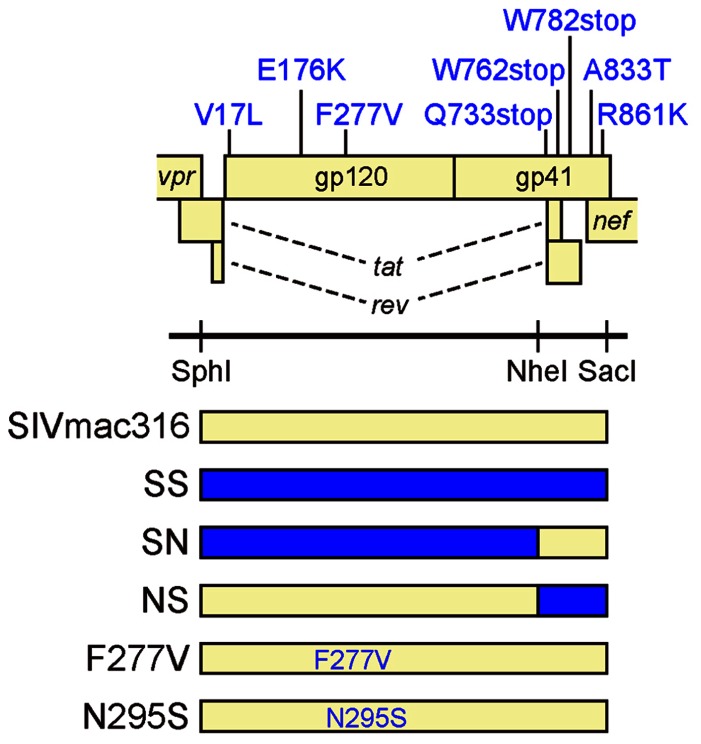
**Construction of infectious SIV clones with B404-resistant mutations**. The open reading frames of the SIV genome are shown along with the Env substitutions in the B404-resistant variant typical to P26B404I. Full-length SIV clones were constructed from parental SIVmac316 by replacing the *env* region with that of the B404-resistant variant (blue) using *Sph*I, *Nhe*I, and *Sac*I sites. The resultant virus SS contains all of the Env substitutions present in P26B404I. Viruses SN and NS contain substitutions in gp120 and gp41, respectively. Point mutants F277V and N295S were constructed by inserting the substitutions F277V and N295S into SIVmac316, respectively.

**FIGURE 3:**
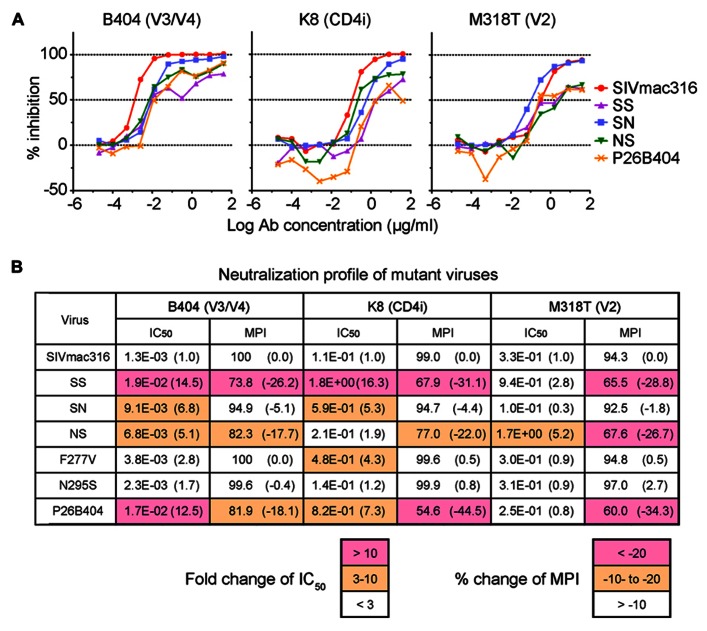
**Sensitivities of viruses with B404-resistant mutations to neutralization by MAbs**. **(A)** Sensitivities of mutant viruses SS, SN, and NS to neutralization by MAbs Fab-B404 (anti-V3/V4), Fab-K8 (anti-CD4i), and M318T (anti-V2) are shown. Neutralization of parental SIVmac316 and B404-resistant P26B404 is also shown as controls sensitive and resistant to neutralization, respectively. **(B)** The sensitivities to neutralization are represented by values for IC_50_ (μg/ml) and MPI (%). Viruses were examined for their sensitivities to neutralization by MAbs Fab-B404, Fab-K8, and M318T in TZM-bl cells. The IC_50_ and MPI values were determined using Prism 5. The fold-change of IC_50_ was calculated by dividing the IC_50_ value by that of the parental SIVmac316. Percent change of MPI was calculated by subtracting the MPI value from that of SIVmac316. These changes are shown in parentheses and significant changes are indicated by magenta (IC_50_: >10; MPI: < -20) and orange (IC_50_: 3–10; MPI: -20 to -10) highlighting.

These mutant viruses were examined for their sensitivity to neutralization by three MAbs B404 (V3/V4 conformational), K8 (CD4i), and M318T (V2). The neutralization of SS that contain the entire *env* region from P26B404I was similar to those of P26B404, indicating that the *env* region is responsible for the resistance to neutralization (**Figure 3A**). Recombinants SN and NS, which have substitutions in gp120 and gp41 from P26B404I, respectively, showed varying degrees of resistance. The IC_50_ values of SN and NS against B404 were intermediates between the parental SIVmac316 and the neutralization-resistant P26B404. Maximal inhibition reached a plateau at 73.8, 82.3, and 81.9% in SS, NS, and P26B404, respectively, but the MPI value of SN (94.9%) was close to that of SIVmac316 (100%; **Figure 3B**). Neutralization resistance to anti-CD4i MAb K8 was characterized by decreases in the IC_50_ value of SN and the MPI of NS. Neutralization by anti-V2 MAb M318T was even enhanced in SN, although NS showed the resistance comparable to those of SS and P26B404. The decreases in MPI values were commonly observed for the neutralization of NS by the three MAbs (**Figure 3B**). Resistance to neutralization was not significantly detected by the point mutants F277V and N295S, except for the neutralization of F277V by K8 (4.3-fold decrease of IC_50_ value). These results indicated that the entire *env* region, including substitutions in both gp120 and gp41, was responsible for the full-resistance of P26B404 to neutralization. The decrease of MPI values for NS suggested that truncation of gp41 by the Q733stop substitution, the first major substitution in viral evolution, was important to escape from the neutralizing antibodies.

### INCREASED INFECTIVITY FOR HUMAN CELLS BY SIV WITH A TRUNCATED gp41

Truncation of gp41 in SIV is associated with the adaptation to human cells (Hirsch et al., 1989; Kodama et al., 1989), which may partially contribute to neutralization resistance (Yuste et al., 2005). To explore the mechanism of neutralization resistance of P26B404, the infectivity of recombinant viruses was analyzed by determining the TCID_50_ values of virus stocks prepared by transfection of 293T cells (**Table 2**). The TCID_50_ values in all the human cells tested were significantly higher for SS and NS viruses with truncated gp41 than parental SIVmac316 and SN, in which gp41 is intact. In particular, NS showed a striking increase in TCID_50_ values, which were 7,100-, 1,000-, and 140-fold higher than those of parental SIVmac316 in PM1, PM1/CCR5, and TZM-bl cells, respectively. These results indicate that truncation of gp41 caused by the Q733stop substitution increases viral infectivity for human cells.

**Table 2:** Infectivity* of viruses with substitutions from P26B404.

Viruses	PM1	PM1/CCR5	TZM-bl
SIVmac316	4.2E+02 (1.0)	1.4E+03 (1.0)	9.6E+04 (1.0)
SS	2.9E+05 (710)	4.7E+05 (350)	6.3E+06 (66)
SN	2.0E+03 (4.8)	8.4E+03 (6.2)	2.9E+05 (3.1)
NS	2.9E+06 (7,100)	1.4E+06 (1,000)	1.4E+07 (140)

*Infectivity is shown by the TCID_50_/ml values of the viruses, which were prepared by transfection of 293T cells, in PM1, PM1/CCR5, and TZM-bl cells. The fold-change, which was calculated by dividing the mutant TCID_50_/ml value by that of the parental SIVmac316, is shown in the parentheses.

**FIGURE 4:**
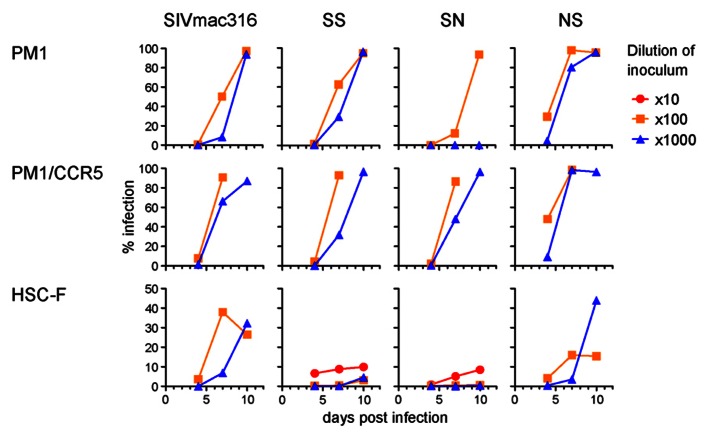
**Replication of B404-resistant viruses in human and macaque cells**. Frequencies of infected cells at 4, 7, and 10 days post-infection are shown. PM1 and PM1/CCR5 human T cells and HSC-F cynomolgus macaque T cells were infected with the various dilutions of SIVmac316, SS, SN, and NS. Infected cells were detected using flow cytometry after intracellular staining by B450 (anti-p27 Fab).

To compare viral infectivity in human and macaque cells, viral infection was monitored after inoculation of PM1 and PM1/CCR5 human cells and the HSC-F cynomolgus macaque cell line with varying dilutions of virus stocks (**Figure 4**). Consistent with the TCID_50_ analysis, a higher frequency of infected cells was detected earlier in PM1 and PM1/CCR5 cells inoculated with NS than the parental SIVmac316. In contrast, SN showed decreased infectivity in PM1 and PM1/CCR5 cells, apparently because PM1 cells were not infected by a 1,000-fold diluted SN stock. Although the TCID_50_ values of SS were much higher than those of SIVmac316, the replication kinetics of SS were similar to those of SIVmac316 in PM1 and PM1/CCR5 cells. These results suggest that gp41 truncation increases infectivity for human cells and that the substitutions in gp120 of P26B404I are associated with slow and poor replication compared with that of SIVmac316.

Infectivity for macaque cells was more significantly affected than that for human cells by the substitutions in gp120 of P26B404I (**Figure 4**, lower panels). Infected cells were detected in HSC-F cells inoculated with 1,000-fold diluted virus stocks of SIVmac316 and NS, but viral infection in HSC-F cells was limited to a low frequency even by inoculation with 10-fold diluted virus stocks of SS and SN. Truncation of gp41 did not significantly affect replication in HSC-F macaque cells, although truncation of gp41 was disadvantageous for replication in primary T cell cultures from macaques (Hirsch et al., 1989; Kodama et al., 1989).

These results demonstrate that gp41 truncation strikingly increases infectivity for human cells, but not for macaque cells, and that the substitutions in gp120 decrease infectivity in human and macaque cells. Truncation of gp41, which conferred extremely high infectivity for PM1/CCR5 cells, may be the first step to escape from neutralization and the substitutions in gp120 may be the second step to replicate in the presence of high concentration of B404.

### INCREASED INCORPORATION OF Env INTO VIRIONS IN SIV WITH TRUNCATED gp41

**FIGURE 5:**
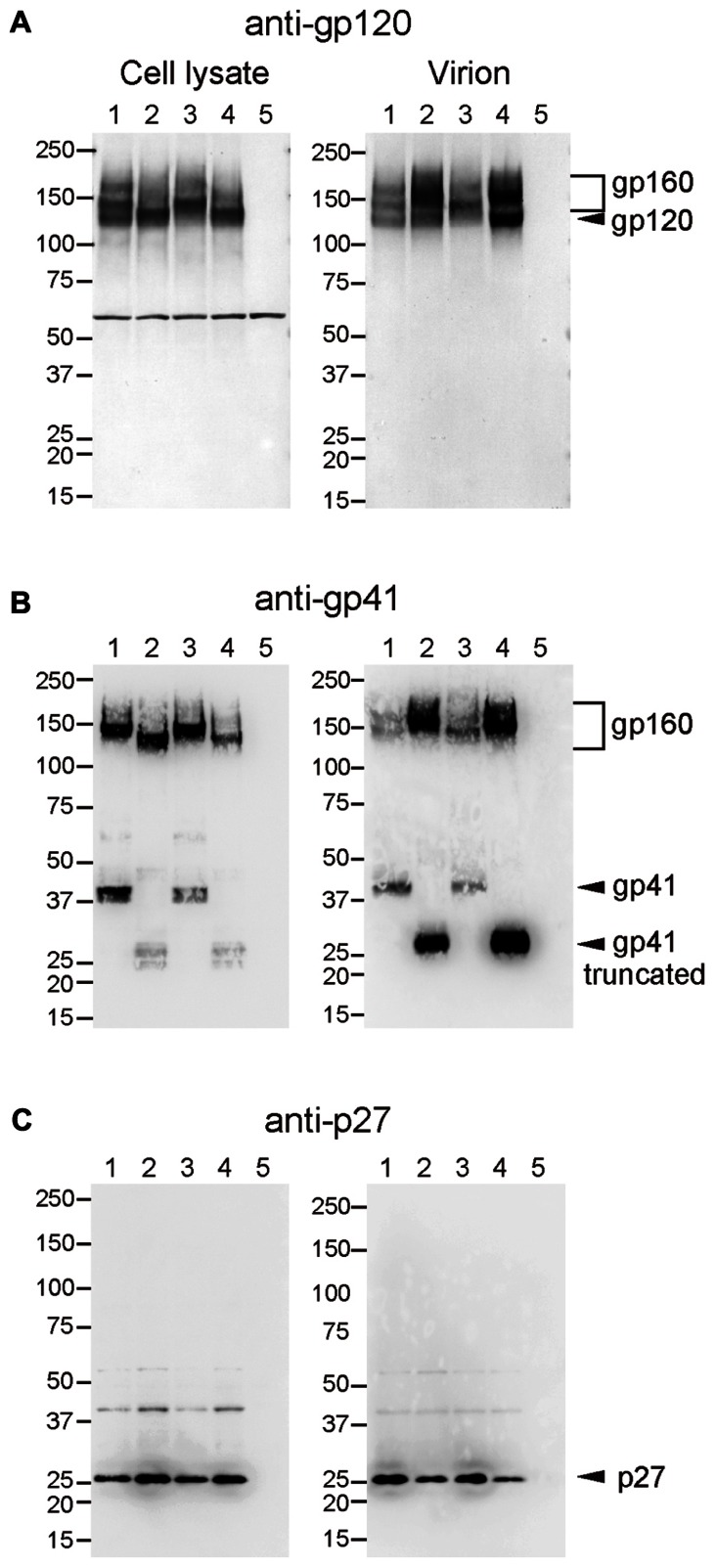
**Effect of B404-resistant mutations on the incorporation of Env into virions**. Cell lysates (left panels) and virions (right panels) of SIVmac316 (lane 1), SS (lane 2), SN (lane 3), NS (lane 4), and mock-infected control (lane 5), which were prepared from transfected 293T cells, were analyzed by Western blotting. Viral proteins gp120 **(A)**, gp41 **(B)**, and p27 **(C)** were detected using MAbs M318T (anti-gp120 V2), B408 (anti-gp41 cluster I), and B450 (anti-p27), respectively.

Incorporation of Env into virions was examined using these recombinant viruses, because increased infectivity by gp41 truncation was suggested to be associated with the Env content of virions (Manrique et al., 2001; Zhu et al., 2003, 2006; Yuste et al., 2004, 2005). Analysis of viral proteins in cells and supernatants from transfected 293T cells revealed that incorporation of Env into virions was significantly high in SS and NS viruses with the Q733stop substitution (**Figure 5**). MAb to gp120 showed a higher amount of gp120 and gp160 in virions from SS and NS than those from SN and the parental SIVmac316, although the production of Env proteins in the transfected cells was at the same level among all the viruses (**Figure 5A**). MAb to gp41 also demonstrated that truncated gp41 was more abundant in virions compared with full-length gp41 (**Figure 5B**). Semi-quantification by densitometric scanning of gp41 and p26 images suggested that the levels of gp41 amount per virion in SS and NS were 12- and 44-fold higher than that of SIVmac316, respectively, after adjusting virion numbers using the p26 amounts. In contrast to the increased amount of Env proteins in virions from viruses with truncated gp41, the level of Gag p27 in virions was low in SS and NS compared with those in SN and SIVmac316 (**Figure 5C**). This indicates that the Env content per virion, which was normalized by the amount of p27, was significantly high in viruses with truncated gp41. These results suggest that truncation of gp41 by the Q733stop substitution enhances incorporation of Env into virions.

### NEUTRALIZATION RESISTANCE OF SIV WITH TRUNCATED gp41 IN MACAQUE CELLS

**FIGURE 6:**
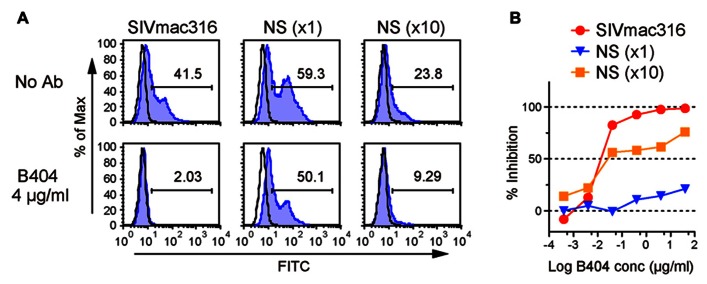
**Contribution of gp41 truncation to neutralization resistance in infection of macaque cells**. **(A)** Infection of HSC-F cells in the presence (4 μg/ml) or absence of Fab-B404 is shown. HSC-F cells were inoculated with undiluted SIVmac316 and NS, and 10-fold diluted NS. Viral infection was monitored 4 days post-infection using flow cytometry after intracellular staining by B450 (anti-p27 Fab). **(B)** Neutralization of SIVmac316 and NS by B404 in HSC-F cells are shown. Percent inhibition was calculated by comparison with the infected culture in the absence of B404.

The analysis of infectivity of recombinant viruses suggested that the resistance to neutralization by truncation of gp41 might be due to adaptation to human cells. To examine this hypothesis, sensitivity to neutralization by B404 was determined in HSC-F macaque cells using SIVmac316 and NS, which showed similar infectivity for HSC-F cells (**Figure 4**). In flow cytometric analysis, infection in the presence or absence of B404 demonstrated that the high sensitivity of SIVmac316 and resistance of NS to neutralization were maintained in HSC-F cells (**Figure 6**). The frequency of infected cells decreased from 41.5% to the background level (2.03%) in inoculation with the undiluted stock of SIVmac316. In contrast, infection with NS, even with a 10-fold diluted virus stock, was significant in HSC-F cells in the presence of B404 (**Figure 6A**). Neutralization of NS in HSC-F cells was characterized by a decrease in maximal inhibition (**Figure 6B**), which was also observed in TZM-bl cells (**Figure 3A**). The magnitude of resistance of NS to B404 was greater when infection was performed using the undiluted stock compared with the 10-fold diluted stock, raising the possibility that B404 did not inhibit infection with a high titer of viruses. However, the resistance of NS was shown by infection with a low titer of NS, in which the frequency of infected cells in the absence of B404 (23.8%) was lower than infection with undiluted SIVmac316 (41.5%). Further, immunoblotting analysis revealed that the amount of virions was higher in the virus stock of SIVmac316 than that of NS (**Figure 5**).

These results indicate that gp41 truncation by the Q733stop substitution contributes to neutralization resistance of viruses in macaque cells. This suggests that the resistance to neutralization by truncation of gp41 is not due to the adaptation to human cells. The Q733stop substitution, the first major mutation during passages in the presence of B404, might be selected because it facilitates adaptation of virus to human cells and imparts resistance to antibody.

## DISCUSSION

In the present study, truncation of the cytoplasmic tail of gp41, which was caused by the Q733stop substitution in Env, was the first major mutation detected during passage of SIV in the presence of the neutralizing antibody B404. Analysis of recombinant viruses suggested that the gp41 truncation was selected by their resistance to neutralizing antibody, which was characterized by the decrease of maximal inhibition compared with viruses with intact gp41, and increased infectivity for human cells. The premature stop codon in the gp41 cytoplasmic region was frequently detected in SIV strains propagated in human cell culture *in vitro*, such as the original SIVmac316 clone, SIVmac1A11 and 17E-Fr (Hirsch et al., 1989; Kodama et al., 1989; Mori et al., 1992; Bonavia et al., 2005; Vzorov et al., 2005). The truncation of gp41 is considered as an adaptation of SIV to replication in human cell culture, because the premature stop codon rapidly reverted to express full-length gp41 after infection of rhesus primary cell culture *in vitro* and rhesus macaques *in vivo* (Hirsch et al., 1989; Kodama et al., 1989). Mutant viruses harboring the gp41 truncation showed increased infectivity for human cells, although the effects on infectivity varied depending on the SIV strain and the length of the gp41 truncation (Manrique et al., 2001; Yuste et al., 2004, 2005; Vzorov et al., 2005, 2007). The enhancement effect of gp41 truncation on incorporation of Env into virions, which were demonstrated by quantification of viral proteins in virions (Yuste et al., 2004) and electron tomography analysis of Env trimers on virions (Zhu et al., 2003, 2006), was partly associated with the increased infectivity caused by gp41 truncation (Manrique et al., 2001; Yuste et al., 2004, 2005). Because expression of Env on the cell surface is regulated by the cytoplasmic domain of gp41, truncation of gp41 may increase Env density on both cells and virions (LaBranche et al., 1995; Berlioz-Torrent et al., 1999; Postler and Desrosiers, 2013). Consistent with these studies, infectivity for human cells and Env incorporation into virions was enhanced by gp41 truncation in the present study. Although the mechanism responsible for increasing viral infectivity caused by gp41 truncation remains unclear, the high virion Env content may contribute to the efficient replication of viruses with truncated gp41 in human cells.

The effect of gp41 truncation on susceptibility to antibody-mediated neutralization is controversial, perhaps due to the SIV strains used for the analyses. Because most of prototypic SIV clones with truncated gp41 were macrophage-tropic, CD4-independent, and neutralization-sensitive (Mori et al., 1992; Bonavia et al., 2005; Vzorov et al., 2005), the truncation of gp41 was assumed responsible for the high sensitivity to neutralization. However, the resistance to neutralization by gp41 truncation was shown using the E767stop mutant of SIVmac316 (Yuste et al., 2005). This is consistent with our results using SIVmac316 harboring the Q733stop substitution, indicating that gp41 truncation contributes to resistance of SIVmac316 to neutralization. The increased infectivity of viruses with gp41 truncation in human cells may partially play a role in resistance by overcoming antibody-mediated neutralization via efficient attachment and entry of viruses to cells. However, we showed that gp41 truncation was also associated with neutralization resistance in macaque cells, in which gp41 truncation did not significantly affect infectivity. This suggests that the increased infectivity in human cells does not significantly affect the neutralization resistance of viruses with truncated gp41. As shown by provision of excess Env in trans, high Env content in virions may be critical for antibody-mediated neutralization (Yuste et al., 2005). Further studies will be required to understand the mechanism of resistance to neutralization conferred by gp41 truncation.

In the present study, we demonstrated that truncation of the cytoplasmic tail of gp41 contributes to resistance to antibody-mediated neutralization. Although non-human primate models of SIV infection are commonly used to estimate vaccine efficacy, the lack of broadly neutralizing MAbs has hampered development of antibody-based vaccine candidates in an SIV-macaque model. The broadly neutralizing MAb B404, which neutralizes multiple, diverse SIV isolates (Kuwata et al., 2013), is a useful tool for understanding the mechanism of neutralization in an SIV-macaque model and will contribute to the development of HIV-1 vaccines.

## Conflict of Interest Statement

The authors declare that the research was conducted in the absence of any commercial or financial relationships that could be construed as a potential conflict of interest.
